# Osteocyte-mediated mechanical response controls osteoblast differentiation and function

**DOI:** 10.3389/fphys.2024.1364694

**Published:** 2024-03-11

**Authors:** Heather VerValin Buck, Joseph Paul Stains

**Affiliations:** School of Medicine, University of Maryland, Baltimore, MD, United States

**Keywords:** osteoblast, differentiation, osteoblastogenesis, mechanical loading, sclerostin, Wnt, osteocyte

## Abstract

Low bone mass is a pervasive global health concern, with implications for osteoporosis, frailty, disability, and mortality. Lifestyle factors, including sedentary habits, metabolic dysfunction, and an aging population, contribute to the escalating prevalence of osteopenia and osteoporosis. The application of mechanical load to bone through physical activity and exercise prevents bone loss, while sufficient mechanical load stimulates new bone mass acquisition. Osteocytes, cells embedded within the bone, receive mechanical signals and translate these mechanical cues into biological signals, termed mechano-transduction. Mechano-transduction signals regulate other bone resident cells, such as osteoblasts and osteoclasts, to orchestrate changes in bone mass. This review explores the mechanisms through which osteocyte-mediated response to mechanical loading regulates osteoblast differentiation and bone formation. An overview of bone cell biology and the impact of mechanical load will be provided, with emphasis on the mechanical cues, mechano-transduction pathways, and factors that direct progenitor cells toward the osteoblast lineage. While there are a wide range of clinically available treatments for osteoporosis, the majority act through manipulation of the osteoclast and may have significant disadvantages. Despite the central role of osteoblasts to the deposition of new bone, few therapies directly target osteoblasts for the preservation of bone mass. Improved understanding of the mechanisms leading to osteoblastogenesis may reveal novel targets for translational investigation.

## 1 Introduction

In the healthy skeleton, bone must respond to environmental cues to accommodate the organism’s needs. This is easily demonstrated in athletes, where the results of repetitive high-intensity loading are clear. Both male and female collegiate tennis players, for example, develop significantly increased bone mineral density in the dominant arm, as compared to the contralateral arm (McClanahan et al., 2002). Significant differences between an athlete’s dominant and non-dominant arm can also be observed in a range of sports, like baseball, golf, and volleyball. Through training and competition, athletes expose their skeletons to repeated and intense strain; their bones respond to increased strain by upregulating anabolic bone processes, like osteoblast differentiation, extracellular matrix deposition, and mineralization to strengthen the bone and resist fracture. This increase in bone mass is accomplished through the close interplay of various bone-resident cells, namely, osteoblasts, osteocytes, and osteoclasts. Generally, osteoblasts deposit and mineralize the collagen-rich extracellular matrix that will become new bone. Osteocytes receive mechanical and hormonal signals and transduce these cues among themselves and to osteoblasts and osteoclasts to coordinate bone deposition and resorption. Osteoclasts resorb mineralized bone and digest the collagenous extracellular matrix. Osteoclasts and osteoblasts are on-demand cells that form when they are needed and later become quiescent or undergo apoptosis. In contrast, osteocytes are long-lived cells that mediate the activation of osteoblast and osteoclasts. This review will discuss the bone anabolic response to mechanical load, with a focus on how osteocytes sense and respond to loading to affect osteoblast differentiation.

### 1.1 Osteoblasts

Osteoblasts are cuboidal cells found on bony surfaces, where they are the primary producer and depositor of the extracellular matrix that will become mineralized bone (Mohamed, 2008). They arise from mesenchymal progenitor cells, which can differentiate into a range of cells such as adipocytes, chondrocytes, and myocytes. They express mechanoresponsive calcium channels, like Piezo1, and have been shown *in vitro* to respond to tensile load with increased alkaline phosphatase expression and mineralization (Liu et al., 2022).

Following local bone resorption by osteoclasts, osteoblasts are recruited to the newly exposed bone surface where they begin to secrete collagen and other extracellular matrix proteins at their apical face (Blair et al., 2017). This newly formed extracellular matrix, termed osteoid, is an arrangement of primarily type I collagen trimers, which are highly interlinked, along with other extracellular matrix proteins like osteopontin, osteocalcin, bone sialoprotein, and osteonectin (Orgel et al., 2001). Osteoid also contains other organic components, like embedded latent growth factors, and provides the lattice into which mineralization will occur (Linkhart et al., 1996).

The inorganic component of bone, hydroxyapatite, is comprised of phosphate and calcium, vital minerals found in circulation. Following matrix deposition, osteoblasts support hydroxyapatite crystal nucleation by expressing proteins such as alkaline phosphatase, which provide the appropriate inorganic phosphate and hydrolyze the mineralization inhibitor, pyrophosphate (Andrade et al., 2019), and bone sialoprotein, which stimulates hydroxyapatite nucleation (Hunter et al., 1996). The presence of fibrillar collagen, alkaline phosphatase, and the absence of endogenous inhibitors of mineralization seem to be the key combination that permits this tissue to mineralize (Murshed et al., 2005). After mineralization, osteoblasts either become entrapped into the accumulating bone matrix and differentiate into osteocytes, become quiescent bone lining cells, or undergo apoptosis (Dallas and Bonewald, 2010).

### 1.2 Osteocytes

Osteocytes, which are found throughout mineralized bone in most vertebrates (Shahar and Dean, 2013), are the primary integrator of mechanical signaling (Schaffler et al., 2014), utilizing their distinctive location encased in mineralized bone and their neuron-like morphology to detect mechanical load in the surrounding bone. Osteocytic cell bodies are located within ovular chambers, called lacunae, and their long, thin processes reside in a geometrically complex, three-dimensional series of tunnels, called canaliculi. This series of chambers and tunnels, the lacunar-canalicular network, is highly interconnected and provides a conduit for direct cell-to-cell communication amongst osteocytes and between osteocytes and other bone resident cells, mediated by gap junctions (Moorer et al., 2017). Surrounding the osteocytes, the fluid-filled space within the lacunar-canalicular system contains extracellular matrix elements like collagen, glycocalyx (Burra et al., 2011), and perlecan (Thompson et al., 2011), which is believed to be a primary tethering protein, critical for load response (Wang et al., 2014).

When mechanically stimulated, osteocytes express many bone anabolic effectors, like WNT, nitric oxide, and PGE2, which act to upregulate the β-catenin pathway; unloaded osteocytes express catabolic signals like RANKL and the β-catenin pathway inhibitor sclerostin. Osteocytes are terminally differentiated osteoblasts (Mullen et al., 2013), and while the mechanism of their encapsulation within bone matrix has not been fully clarified (Franz-Odendaal et al., 2006; Robling and Bonewald, 2020), they are often distinguished from osteoblasts by their expression of markers such as DMP1, FGF23, podoplanin, and sclerostin (Delgado-Calle and Bellido, 2022).

### 1.3 Osteoclasts

Osteoclasts are large, multinucleated cells derived from macrophages and are the primary facilitator of bone resorption. They originate from hematopoietic stem cells and their differentiation is promoted by the binding of RANKL (receptor activator of NFκB ligand) (Yasuda et al., 1998) to its cognate receptor (RANK) on the surface of osteoclast progenitors. Osteocytic RANKL expression is increased during unloading, supporting increased osteoclastogenesis and bone resorption during disuse (Ono et al., 2020). Emerging data suggest that osteoclasts may also directly respond to mechanical stimulation (Dsouza and Komarova, 2023), but the way in which this response may participate in anabolic processes is not settled.

To remove mineralized bone, osteoclasts adhere to the bone surface, form an actin ring, and vectorially secrete acidic vesicles into the bone-facing extracellular milieu (Teitelbaum, 2011). The accumulation of hydrogen protons and proteolytic enzymes on the bone-facing surface dissolve mineralized bone and extracellular matrix, liberating calcium and phosphate ions and forming a resorption pit (Han et al., 2019).

### 1.4 Osteoprogenitors and the osteoblast cell lineage

A broad group of mesenchymal progenitor cells, commonly found in the bone marrow cavity, around vasculature, or in the fibrous periosteal and endocortical membrane surfaces that line cortical bone, give rise to skeletally associated cells including chondrocytes, adipocytes, and osteoblasts (Kurenkova et al., 2020; Matsushita et al., 2020). Lineage allocation of these progenitor cells is mediated by specific cues and activators. Canonically, the transcription factor Sox9 is required for the differentiation of chondrocytes (Wheatley et al., 1996), cells necessary for growth, limb development, and cartilage maintenance. Differentiation into adipocytes, cells that support metabolism and lipid storage, is supported by the transcription factor PPARγ (Lefterova et al., 2008), while osteoblast differentiation requires the transcription factors RUNX2 (Drissi et al., 2000) and Osterix (Nakashima et al., 2002).

There are several influences that can bias mesenchymal progenitor cells towards osteoblast or adipocyte differentiation. For example, consistent administration of a high-fat diet increases the relative differentiation of progenitor cells into adipocytes over osteoblasts (Parhami et al., 2001), while osteoblastogenesis is favored after mechanical load (Sen et al., 2008). The stiffness of the extracellular matrix surrounding bone-resident cells is also believed to influence differentiation and anabolic response. It has been demonstrated *in vitro* that mesenchymal progenitor cells are more likely to differentiate into the osteogenic lineage (Engler et al., 2006) and that expression of osteoblastic deposition markers (Zhang et al., 2017) increase in the presence of a relatively stiff extracellular matrix.

Even within the osteoblast lineage, varied cues can activate distinct progenitor populations to differentiate into bone-depositing osteoblasts. For example, discrete groups of skeletal progenitor cells may be recruited from the periosteal or endosteal compartments in response to fracture or mechanical loading (Atria and Castillo, 2023), though this is still an area of emerging research.

### 1.5 Mechanical load: Tissue and cellular responses

Like many other physiological systems, bone mass is regulated around a homeostatic set point. Mechanical loading, such as from customary physical activity to which an organism is acclimated, will be within the homeostatic range and insufficient to induce a net change in bone mass (Robling and Turner, 2009). This broad range of mechanical loading is referred to as the “adapted window” in Harold Frost’s mechano-stat theory (Frost, 2003) and is also known as the lazy zone. This level of load will be different for every organism and will adapt to the organism’s activity level and peak loading forces. For example, an individual who regularly strength trains will accumulate more skeletal mass to adapt to increased load, but may not receive additional anabolic benefit from exercising with a weight to which they have habituated, even if that weight would be sufficient to drive deposition in a novice weightlifter.

The mechanical stimulation experienced by a population of osteocytes (known as strain) is in part controlled by the strength and resistance of their surrounding bone. Relatively high loads cause shape changes in the bone, which shifts fluid within the lacunar-canalicular system, stimulating osteocytes (Price et al., 2011). These osteocytes locally induce osteoblastogenesis and increased bone mass. After mineralization, the same level of load may no longer be sufficient to cause deflection of the thicker, more resistant bone, resulting in reduced strain perceived by osteocytes. Consistent lack strain leads to resorption and thinner, more easily deflectable bones that will become more responsive to a given load. This process of continual readjustment tunes bone to the changing needs of the organism (Christen et al., 2014).

Load above the homeostatic range activates osteoblastogenesis (Turner et al., 1998) and leads to the deposition of new bone; this range is known as the bone overload zone. Deposition does not occur globally, however, and is generally proportional to the amount of experienced strain (Robling et al., 2002). In contrast to mechanical loading, unloading or disuse refers to a significant loss of mechanical stimuli and may be the result of local immobilization after fracture (Ceroni et al., 2012) or paralysis (Dionyssiotis et al., 2007), or may affect the total organism, as during bedrest (Leblanc et al., 1990) or microgravity (LeBlanc et al., 2000). Sustained unloading leads to an increase in osteoclast number and activity (Ishijima et al., 2001) and decreased osteoblastogenesis and deposition (Dufour et al., 2008), resulting in resorption and a net loss of bone mass. For either strenuous activity or disuse, bone adapts to the loading environment to which it is routinely exposed.

When sufficient force is applied to bone it flexes, causing fluid within the lacunar-canalicular system to be displaced (Piekarski and Munro, 1977). The passage of fluid over the surface of the osteocyte creates shear stress against the cell, while movement of the tethering proteins that link the osteocyte to the bone extracellular matrix is thought to amplify the mechanical stimulation of the osteocyte (You et al., 2001). Mechanical signals are transmitted into the osteocyte through factors such as force-gated ion channels like Piezo1 (Li et al., 2019) and TRPV4 (Lyons et al., 2017; Williams et al., 2020), which allow for rapid calcium influx and deformation of the cytoskeleton (Han et al., 2004; Lyons et al., 2017) and primary cilia (Lee et al., 2015), which engage a range of anabolic signaling elements in the local area, including nitric oxide (Klein-Nulend et al., 1995), reactive oxygen (Lyons et al., 2017), PGE2 (Jiang and Cheng, 2001), IGF1 (Lean et al., 1996), BMP-2 (da Silva Madaleno et al., 2020) and WNTs (Du et al., 2019). Additionally, loading causes osteocytes to reduce expression of proteins, like sclerostin and DKK1. Ultimately, many of these signaling factors participate in the β-catenin pathway.

In response to mechanical loading, osteocytes seem to respond in a binary manner with respect to calcium influx (Schaffler and Kennedy, 2012), with each osteocyte in either a ‘loaded’ or ‘non-loaded’ state. One loading event does not stimulate every osteocyte, however; osteocytes in regions of bone experiencing the highest mechanical strain are more likely to be stimulated, and osteocytes in low strain areas are less likely to be stimulated. The magnitude of the loading event can increase the percentage of osteocytes in a given anatomic region that go into the ‘loaded’ state, with strain frequency being the largest determinant of successful stimulation (Lewis et al., 2017). The type of force applied, like tensile, compressive, or stretch, can also influence the molecular response (Josephson and Morgan, 2023). The overall distribution and number of these ‘loaded’ and ‘non-loaded’ osteocytes contributes to the tuning of local bone mass.

## 2 Molecular signals mediate the cellular effects of mechanical loading

### 2.1 Sclerostin

Sclerostin is a glycoprotein secreted by mature osteocytes that can signal to a variety of cells and tissues (Weivoda et al., 2017) and also control deposition and resorption through regulation of bone-resident cells. Disruptive variants or genetic knockout of the *Sost* gene that encodes sclerostin are sufficient to produce a phenotype characterized by excessive skeletal deposition in both humans (Brunkow et al., 2001) and mice (Li et al., 2008).

An osteocyte in the lazy zone, or one experiencing disuse, will constitutively secrete sclerostin, signaling that bone formation is not needed. In response to mechanical loading and other bone anabolic cues, sclerostin protein is reduced and bone formation is unleashed.

When secreted by osteocytes, sclerostin suppresses osteoblast differentiation and promotes osteoclast number and activity (Suen and Qin, 2016). Additionally, sclerostin may bind to osteoblasts, preventing their differentiation into osteocytes (Atkins et al., 2011). Sclerostin also functions as an endocrine signal, perhaps facilitating glucose and fatty acid availability for new bone formation when sclerostin expression is low (Riddle, 2023), but the direct effect of these processes on bone mass have yet to be fully clarified (Fu et al., 2022; Gu et al., 2023).

Sclerostin suppresses the ability of mesenchymal progenitor cells, pre-osteoblasts, and bone lining cells to differentiate into osteoblasts via inhibition of the β-catenin pathway (Rutkovskiy et al., 2016). Sclerostin binds to LRP5/6, where it acts as a competitive inhibitor of WNT family members (Li et al., 2005), like WNT3 and WNT10 (Krishnan et al., 2006). Once sclerostin binds to LRP5/6, LRP5/6 cannot form a complex with Frizzled, and β-catenin becomes ubiquitinated and is degraded by the proteosome (Liao et al., 2022). Other mechanically regulated inhibitors of WNT binding, like DKK1, have been shown to suppress osteoblast differentiation in a similar way (Ke et al., 2012).

In contrast, a decrease in sclerotin abundance allows WNT to bind with LRP5/6, disrupting the β-catenin destruction complex (Stamos and Weis, 2013). Stabilized β-catenin translocates to the nucleus where it acts to support Runx2 and Osx (osterix) expression, leading to osteoblast differentiation and the expression of osteoid-associated genes (Felber et al., 2015). Thus, β-catenin signaling is fundamentally important to increased osteoblast number and subsequent matrix deposition.

In addition to bone formation, sclerostin may also regulate bone resorption. *In vitro*, treatment of osteocyte-like cells with exogenous sclerostin leads to increased RANKL expression (Wijenayaka et al., 2011). Overall, when sclerostin levels are high, osteoblast differentiation and bone formation are inhibited, while osteoclast formation and bone resorption are elevated. High levels of sclerostin, as is typically seen with disuse, is net catabolic to bone.

**FIGURE 1:**
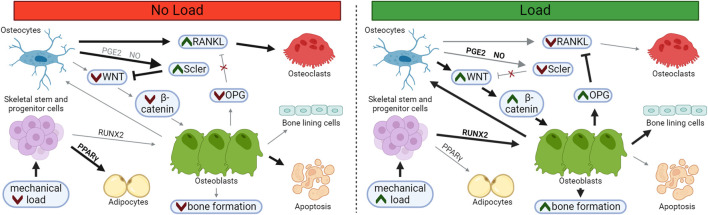
Loading Supports Osteoblast Differentiation and Suppresses Osteoclast Differentiation. In unloaded bone, sclerostin and RANKL expression is upregulated, supporting osteoclastogenesis and resorption. Following load, osteoblast and osteocyte differentiation increases, while osteoblast apoptosis and osteoclast differentiation decreases, leading to bone matrix deposition. Osteoblasts may embed and mature into osteocytes within the newly formed bone or may transition to quiescent bone lining cells on the newly formed bone surface. Bolded arrows and green chevrons indicate upregulated pathways, red chevrons indicate downregulated pathways.

Loss of sclerostin abundance following mechanical load supports a powerful switch from catabolic to anabolic signaling. Short bouts of axial loading within the high-physiological range *in vivo* are sufficient to cause a temporary loss of sclerostin and induce osteoprotegerin (OPG) expression and an increase in bone mass in regions of high strain (Robling et al., 2008). Sclerostin protein undergoes tight, spatial regulation in regions of greatest mechanical strain, depressing bone formation where load is highest and bone is most likely to fail if appropriate adaptation does not occur. (Figure 1). This strain-dependent loss of sclerostin has been demonstrated following fluid shear stress *in vitro* as well (Gould et al., 2021a).

### 2.2 Nitric oxide

Along with many other anabolic signaling molecules, osteocytes release nitric oxide in response to fluid shear stimulation (Klein-Nulend et al., 1995). Nitric oxide is produced by three isoforms (Nos1, Nos2, and Nos3), which are expressed in a wide range of cell types (Riancho et al., 1995), and is sufficient to drive the loss of sclerostin protein *in vitro* (Gould et al., 2021b). Global knockouts of the *Nos3* gene have been shown to result in decreased trabecular bone volume basally in adult mice, while *Nos2* knockout reduces bone mass recovery after tail suspension and reloading (Watanuki et al., 2002). Interestingly, *Nos1* knockout produces increased murine bone mineral density in cortical and trabecular compartments (van’t Hof et al., 2004). While these isoforms convert the same substrates, they have diverse expression profiles, activators, and repressors, leading to disparate functions in bone. Nitric oxide has long been understood to play an intrinsic role in bone homeostasis, but the translational potential of nitric oxide for bone mass has not been clarified (Liu and Rosen, 2021).

### 2.3 PGE2

Like nitric oxide, prostaglandin E2 (PGE2) is also widely expressed across diverse tissues (Li et al., 2007), is produced by osteocytes in response to stimulation via pulsatile fluid flow (Ajubi et al., 1996), and is sufficient to reduce sclerostin protein abundance *in vitro* (Koide et al., 2017). PGE2 has been shown to support fracture healing (Wittenberg and Wittenberg, 1991) and bone formation (Li et al., 2007), and to also support osteoclastogenesis *in vitro* (Li et al., 2000). Despite increasing both resorption and deposition, treatment with PGE2 appears to result in a net increase in bone mass, though persistent, systemic side effects have prevented it from being utilized as a clinical therapy for low bone mass (Hartke and Lundy, 2001).

### 2.4 WNT

Removal of β-catenin pathway inhibitors (e.g., sclerostin, DKK1) is insufficient to stimulate the pathway unless the cognate ligands are also present during anabolic signaling (Tu et al., 2012). WNTs are canonical anabolic activators of the β-catenin pathway (Kim et al., 2013) and osteoblastic expression of WNT family genes increases after load (Holguin et al., 2016). Through WNT expression, additional osteoblasts may be recruited to support deposition following relatively high strain. WNT1 specifically has been demonstrated to be necessary for local anabolic bone response following axial loading (Lawson et al., 2022).

### 2.5 OPG

In addition to stimulating new bone formation, mechanical loading can also inhibit bone resorption. One mechanism of bone mass control is the regulation of osteoprotegerin following anabolic stimuli. Osteoprotegerin (OPG) is a cytokine receptor that is expressed by osteoblasts that can locally moderate osteoclastogenesis (Cawley et al., 2020). It is not membrane-bound, but rather secreted into extracellular space (Boyce and Xing, 2007) where it acts as a decoy RANKL receptor, preventing RANKL from stimulating osteoclastogenesis and resorption (Udagawa et al., 2000). OPG production increases following mechanical strain (Saunders et al., 2006) and is typically detectable in circulation as well as within the bone environment (Tsukasaki et al., 2020).

**FIGURE 2:**
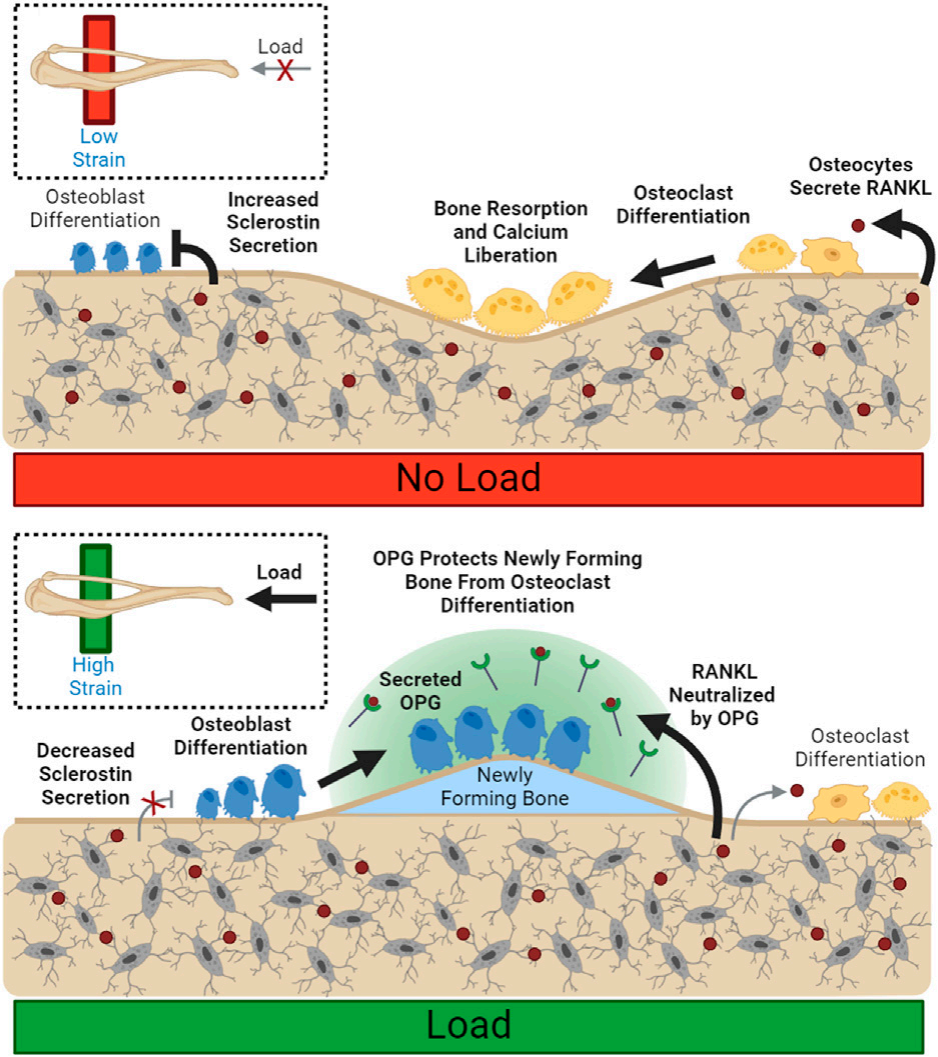
Load Spatially Controls Resorption and Deposition. Unloaded osteocytes secrete sclerostin, preventing osteoblast differentiation. Disinhibited RANKL expression allows for increased osteoclast differentiation and resorption. Load suppresses local sclerostin expression, allowing osteoblast differentiation. Local osteoclastic resorption and differentiation are downregulated through reduced RANKL availability. Bolded arrows indicate upregulated pathways for given conditions.

Conceptually, OPG expression could spatially restrain demineralization in loaded bone and prevent loss of bone mass in specific high-strain regions where new bone is being formed, even during periods of calcium deficit. Biologically active OPG is kept close to the osteoblast membrane, rather than diffused away, through association with osteoblastic surface proteins (Li and Xu, 2020), supporting OPG’s role in local control. (Figure 2). OPG has also been shown to support osteoblastic differentiation *in vitro*, increasing both osteoblast number and upregulation of markers associated with osteoblast maturity, like ALP (Yu et al., 2011).

### 2.6 Embedded growth factors

The majority of organic bone matrix is comprised of type I collagen (Tzaphlidou, 2008), but other components are embedded during deposition, including growth factors such as BMPs, TGF-β and IGF-I (Solheim, 1998). Injury to bone can accumulate in the form of microscopic cracks due to intense, repetitive loading (O’Brien et al., 2003), or can occur acutely, as in fracture. Disruption of the bone results in osteoclastic dissolution of mineralized tissue, releasing these latent osteoid-embedded growth factors into the local environment. These factors may now stimulate osteoprogenitor recruitment and proliferation, osteoblast differentiation, and local bone deposition (Baylink et al., 1993) in order to restore and strengthen the bone.

## 3 Discussion

Osteoporosis, a clinical designation of severe bone loss with greatly increased fracture risk, can result in hospitalization or death, especially in the elderly and frail (Li et al., 2017). There are several classes of clinically available treatments for low bone mass and osteoporosis, some of which effectively increase BMD by targeting the molecular mediators of mechanical loading. Anti-RANKL (denosumab), acts like an OPG mimetic to suppress osteoclastogenesis, and anti-sclerostin (romosozumab) antibody treatments effectively reduce sclerostin bioavailability, reducing osteoclastogenesis and increasing osteoblastogenesis.

Another class of treatment, teriparatide (PTH1-34) and abaloparatide (PTHrP1-34) are synthetic analogs of parathyroid hormone, which has been shown to suppress sclerostin expression at the RNA (Bellido et al., 2005; Wein and Kronenberg, 2018) and protein (Drake et al., 2010; Gould et al., 2021b) level. Parathyroid hormone-analog treatments are highly effective at preserving and restoring bone mass through an increase in osteoblast number and matrix deposition, due in part to β-catenin pathway upregulation (Tian et al., 2011).

The balance of deposition and resorption is necessary for skeletal health, but can be altered in individuals with mineral deficiency, leading to low bone mineral density (BMD) and associated disorders. For example, prolonged vitamin D deficiency can contribute to low bone mass and relatively high proportions of unmineralized osteoid (Francis and Selby, 1997), ultimately leading to rickets or osteomalacia if left untreated. Exercise, as well as dietary vitamin supplementation, have been shown to improve BMD in these patients (Tønnesen et al., 2016).

Like many other tissues, the skeleton follows a ‘use it or lose it’ model, wherein continued disuse leads to a cycle of wasting, decreased capacity, and fragility, especially in aging (Leser et al., 2021), when the balance of deposition and resorption shifts towards net loss of mineralized bone. Bone mass and osteoblast number decrease with age (Almeida, 2012), but study after study (Layne and Nelson, 1999; Mosti et al., 2014; Watson et al., 2018; Holubiac et al., 2022) report the protective benefits of mechanical loading and exercise. Load increases expression of osteoblastogenesis-related signals, like WNTs and OPG, while suppressing sclerostin abundance and osteoclastogenesis-related signals like RANKL. Accordingly, exercise that produces sufficient mechanical load remains the best defense against bone loss.
